# Sequestration of AS-DACA into Acidic Compartments of the Membrane Trafficking System as a Mechanism of Drug Resistance in Rhabdomyosarcoma

**DOI:** 10.3390/ijms140713042

**Published:** 2013-06-25

**Authors:** Marissa Williams, Daniel Catchpoole

**Affiliations:** The Tumour Bank, Children’s Cancer Research Unit, the Children’s Hospital at Westmead, Westmead, NSW 2145, Australia; E-Mail: marissa.williams@sydney.edu.au

**Keywords:** rhabdomyosarcoma, sequestration, membrane trafficking, vesicles, drug resistance, co-inhibitors

## Abstract

The accumulation of weakly basic drugs into acidic organelles has recently been described as a contributor to resistance in childhood cancer rhabdomyosarcoma (RMS) cell lines with differential sensitivity to a novel topoisomerase II inhibitor, AS-DACA. The current study aims to explore the contribution of the endocytic pathway to AS-DACA sequestration in RMS cell lines. A 24-fold differential in AS-DACA cytotoxicity was detected between the RMS lines RD and Rh30. The effect of inhibitors of the endocytic pathway on AS-DACA sensitivity in RMS cell lines, coupled with the variations of endosomal marker expression, indicated the late endosomal/lysosomal compartment was implicated by confounding lines of evidence. Higher expression levels of Lysosomal-Associated Membrane Protein-1 (LAMP1) in the resistant RMS cell line, RD, provided correlations between the increased amount and activity of these compartments to AS-DACA resistance. The late endosomal inhibitor 3-methyladenine increased AS-DACA sensitivity solely in RD leading to the reduction of AS-DACA in membrane trafficking organelles. Acidification inhibitors did not produce an increase in AS-DACA sensitivity nor reduce its sequestration, indicating that the pH partitioning of weakly basic drugs into acidic compartments does not likely contribute to the AS-DACA sequestering resistance mechanism evident in RMS cells.

## 1. Introduction

Rhabdomyosarcoma (RMS) is a malignant childhood tumour originating from striated muscle precursor cells; it is the most common form of soft tissue sarcoma and presents commonly in children and adolescents with a median age of 5 years for cancer diagnosis [1]. Therapy for RMS is administered in a multimodal approach involving surgical removal of the tumour mass followed by chemotherapy and radiotherapy. Two clinical subtypes of RMS exist and can be differentiated by histological determination; Embryonal RMS (ERMS) and Alveolar RMS (ARMS). ERMS is correlated with earlier onset of disease in children in the 2–5 year old age group. This subtype is associated with a good prognosis with a 5 year survival rate of 60% [1–4]. Conversely, the ARMS subtype of RMS has a later onset of disease, generally presenting in adolescence, and is usually associated with a poor prognosis with less than 30% of patients surviving without experiencing relapse [5–7].

Despite the different metastatic nature of the two subtypes, the same chemotherapeutic protocols are still utilised for their treatment. In order to improve and advance therapies for RMS new agents with increased specificity for tumour subtype and progression are required. Treatment for RMS is dominated by the administration of the DNA intercalating agent, doxorubicin, and most notably, topoisomerase poisons such as topotecan and etoposide [6,8]. Recently, our laboratory screened a variety of novel transcriptional inhibitors and topoisomerase poisons to determine the cytotoxic response in RMS cell lines [8]. Cell lines were treated with 9-amino derivatives of the novel DNA-intercalating agent DACA; two RMS cell lines, RD and RH30, which belong to the ERMS and ARMS subtypes respectively, displayed a differential response to the *N*-2[-9dimethyl(amino)ethyl] acridine-4-carboximide derivative AS-DACA, a topoisomerase II inhibitor with solid tumour activity. AS-DACA induced a greater cytotoxic effect in the RH30 cell line compared with the RD cell line; this was expressed by a 190-fold difference in the IC_50_ values of these cell lines with drug treatment [8]. The mechanistic basis for this resistance is uncertain as the RMS cells may possess cellular or molecular properties that render them resistant to anticancer agents through mechanisms that may be either intrinsic or acquired [9,10].

**Figure 1:**
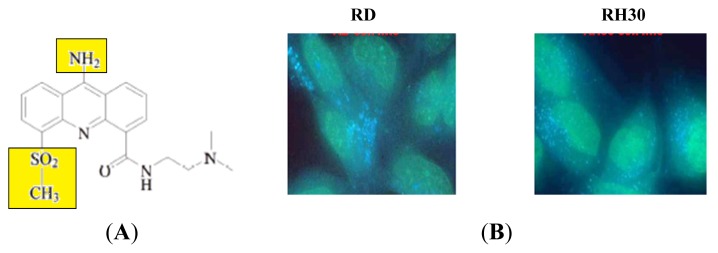
(**A**) Chemical structure of the 9-amino DACA derivative AS-DACA. The highlighted areas confer sites of protonation of the molecular structure in an acidic environment [8]; (**B**) Fluorescence of AS-DACA RMS cells showing nuclear accumulation of the drug as a green emission spectra while the punctate vesicles fluoresce blue, indicating the drug entering compartments with a lower pH [11].

The current study will focus on exploring and expanding the possible drug resistant phenotype apparent in RMS cells. AS-DACA is a 9-amino derivative of the clinical drug candidate DACA (Figure 1A). Its cytotoxicity relies on poisoning of the topoisomerase II enzyme by its DNA intercalating abilities. Its distinctive feature compared to other acridine-4-carboxamide cytotoxins is that its acridine chromophore is weakly alkaline and possesses a pK value of 5.2. This value dictates, that as the environmental pH is lowered below 6 the molecule becomes doubly charged which has great implications in its fluorescent properties [10]. Recent studies by Wolf *et al.* [8] have exploited the pH dependent fluorescence of the drug to visualize its distribution through the RMS cell. This has proved to be particularly useful in studying the cause of differential cytotoxicity between the (relatively) sensitive and resistant cell lines, RH30 and RD, respectively. An interesting outcome of visualizing AS-DACA in these cells was the presence of two different emission colors visualized on one excitation wavelength. A distinct green emission that was seen in the nucleus while small blue vesicles were dispersed around the nucleus (Figure 1B), suggests that the molecule has become charged upon entering an acidic vesicle compartment due to the lower pH in such organelles. The involvement of the endosomal system in sequestering AS-DACA and reducing its potency was further explored by examining the expression of specific markers of organelles belonging to this pathway [11]. These initial findings strongly suggest the involvement of the endosomal pathway in the observed resistance phenotype by the sequestration of AS-DACA into acidic compartments.

We hypothesize that the decreased sensitivity to the drug AS-DACA in RMS cells is due to sequestration of the drug into acidic vesicles of the endosomal pathway. The identification of the specific organelle altered in the resistance apparatus has not been accomplished and this question will form the basis of this investigation. To further characterise the point of resistance in the endosomal pathway, inhibitors of specific components of receptor mediated endocytosis will be employed to determine if they impede endocytosis efficiency and whether they restore AS-DACA sensitivity in RMS cells [12,13]. We will employ four inhibitors affecting different regions of the endosomal pathway in this study: chlorpromazine, bafilomycin A1, chloroquine, and 3-methyladenine. Acidification of the endosomal compartment is enhanced by vacuolar H^+^-ATPase “pumps”, which are inhibited by bafilomycin A1 and chloroquine, and will inhibit components of endosomal function which may be integral to RMS resistance phenotypes [14–16]. Uptake and entry into the endocytic pathway is initiated via clathrin-coated pits, and these will be inhibited by chlorpromazine, which will subsequently reduce the amount of vesicles and recycling routes, thereby sensitizing the cells to AS-DACA [17]. The PI3-Kinase inhibitor, 3-Methyladenine, impedes the progression of late endocytic events and should sensitize RMS cells to AS-DACA [18–20]. We report the inhibitory effects of these inhibitors on the cytotoxic profile of AS-DACA in RMS cells, the intracellular distribution of AS-DACA, and the expression of endosomal proteins in RMS cell lines with treatment of AS-DACA and inhibitors.

## 2. Results and Discussion

### 2.1. Effect of Endocytic Trafficking Inhibitors on AS-DACA Sensitivity in RMS Cells

#### 2.1.1. RMS Cell line Cytotoxic Response to AS-DACA

The observed relative differential in cytotoxic response between the RD and RH30 RMS cell lines to the topoisomerase II inhibitor AS-DACA was confirmed using MTT cell viability assays following 72 h of treatment. These results (Table 1, Line 1) indicated IC_50_ values between the RD and RH30 cell line were approximately 24-fold different with the RH30 cell line being more sensitive to AS-DACA compared to the RD cell line. A two-tailed *t*-test indicated this difference in IC_50_ was statistically significant (*p* < 0.05) (Table 1). Although the cytotoxic differential was not as stark as that in the previous study, the RMS cell lines still exhibited a statistically significant difference in sensitivity to AS-DACA, with RH30 being more sensitive to the drug while RD maintained its relative “resistance” as previously found.

#### 2.1.2. Inhibitor Effect on Cell Lines-Determination of Maximum Tolerated Dose (MTD)

The cell lines were treated with serial 10-fold dilutions of each of four endocytic inhibitors: Chlorpromazine, Bafilomycin A1, Chloroquine and 3-Methyladenine. Dose response curves for both cell lines were established from replicate experiments with IC_50_ values listed in Table 1. Treatment of RD and RH30 cell lines with respect to individual inhibitors and the IC_50_ values were similar for each inhibitor and not statistically significant. The maximum tolerated dose (MTD), values, which were used in latter experiments, were determined to be: 1 μM Chloropromizine, 1 nM Bafilomycin A1, 1 μM Chloroquine and 0.1 mM 3-Methladenine.

A study by Vecauteren *et al.* [21] on the use of endocytic inhibitors in cell cycle studies reported that inhibitors such as Chlorpromazine can have poor specificity on endocytic inhibition, in that they produced various levels of cell viability across the cell lines studied. They conclude that the magnitude of inhibition and cytotoxicity of endocytic inhibitors is highly cell line dependent as exhibited by the differential responses of cell lines to these agents. They highlight that it is important to characterise the effects of endocytic inhibitors on a particular cell line before attempting to analyse the consequences of inhibition. Similarly, here, the cytotoxic effect of each inhibitor used was consistent between cell lines and the MTD between cell lines was constant for each inhibitor treatment. Accordingly, as the inhibitors did not induce a substantial cytotoxic shift between cell lines, the inhibitory action of each separate inhibitor should be considered constant in both cell lines and would allow comparisons of inhibitory effects and their implications between them.

#### 2.1.3. Inhibitor Effect on AS-DACA Cytotoxicity

Endocytic inhibitors have been chosen to impede separate components of the endocytic pathway which have been known to be involved in anti-cancer drug resistance. These inhibitors were included in regular AS-DACA treatment and their influence on AS-DACA sensitivity was observed. Cell lines were treated with the MTD of each inhibitor and 10-fold serial dilutions of AS-DACA simultaneously over 72 h. The IC_50_ concentrations between AS-DACA treatment alone, and with inhibitor co-treatment, were determined and compared using a two-tailed *T*-test.

As most chemotherapeutics are weak bases with a pK values ranging from 7 to 8 [22], the “pH Partitioning Theory” has been the most widely employed explanation for weak base drug sequestration in tumour cells which describes drug diffusion down pH gradients of acidified organelles [23–27]. As this is the accepted mechanism of weak base sequestration in other tumour resistant studies, it was assumed AS-DACA’s sequestration could be facilitated by a similar process at the early endosomal level. Bafilomycin A1 appeared to decrease the sensitivity of AS-DACA in both RD and RH30 by almost half as seen by the IC_50_ values in Table 1. However, these differences were noted to be insignificant by statistical evaluation. Chloroquine co-treatment caused minimal reduction of AS-DACA sensitivity in RD and RH30 cell lines (Table 1). Many studies implicate the pH differentials between acidified organelles and the cytoplasm to account for decreased cellular sensitivity to anticancer agents as a result of their increased vesicular accumulation [27–31]. However, the failure of bafilomycin A1 and chloroquine to increase the cytotoxicity of cells indicates that the disruption of endocytic organelle acidification does not affect the sequestration of AS-DACA in RMS cells, and furthermore, the observed resistance phenotype is not dependent on vesicular acidification. Since AS-DACA cytotoxicity does not appear to be affected by the inhibition of endosomal acidification, it is plausible that RMS cells may sequester AS-DACA through an alternative mechanism of organelle capture.

Increased membrane recycling of endocytic vesicles has been associated with drug resistance and larger numbers of endocytic vesicles have been found in cells with reduced sensitivity to anticancer agents [28,32]. Chlorpromazine’s inhibitory action however, has not been widely implicated in shutting down endocytosis in drug sequestering cancer cells. Despite this, it is appropriate for use in this study due to its inhibition of endocytosis and since it is thought to reduce vesicle number and turnover. Chlorpromazine induced a statistically significant shift in AS-DACA cytotoxicity representing a 1.81-fold increase in sensitivity in the RD cell line compared to the response with AS-DACA treatment alone (Table 1). Chlorpromazine did not affect the sensitivity of AS-DACA in RH30, this cell line being less susceptible to inhibition of endosomal progression. In light of these results, the increase in the sensitivity of RD cells to AS-DACA may be due to the prevention of vesicle formation. However, it cannot be ascertained purely by the modest change in cytotoxicity with Chlorpromazine that increased endosome number or turnover is accountable for AS-DACA resistance in RD cells. This must be confirmed by quantitative studies to determine vesicle number following dose and time-response assays to determine the recycling activities of RMS cells.

Lysosomal compartments have been associated with increased drug uptake in numerous resistant cell lines; they have been reported to have an increased intralumenal area for mechanisms that facilitate the acquisition of anti-cancer drugs from their intracellular target [24,28,33]. 3-Methyladenine has been shown to inhibit the progression of late endosomes to lysosomes by impeding the activity of class 3 PI3-Kinase, an enzyme required for the maintenance of membrane traffic and fusion events [18,34,35]. If AS-DACA is accumulated into vesicles at this late stage of endocytosis it is assumed that impeding the activity of these compartments would result in reduced AS-DACA sequestration. This was observed indirectly by an increased cytotoxic effect. Indeed treatment with 3-Methyladenine also increased the sensitivity of the RD cell line to AS-DACA by approximately 3-fold. This was a statistically significant difference (*p* < 0.05). However, treatment of 3-Methyladenine in RH30 did not increase its sensitivity to AS-DACA (Table 1). This finding implicates the late endosomal or lysosomal compartment in the sequestration and resistance of AS-DACA in the RD cell line.

### 2.2. Effect of Endocytic Trafficking Inhibitors on AS-DACA Distribution in Vesicular Compartments in RMS Cells

#### 2.2.1. Direct Fluorescence of AS-DACA in RMS Cell Lines

The unique fluorescent properties of AS-DACA allowed its intracellular distribution to be monitored in RMS cell lines as previously described [11]. Cell lines were exposed to a final concentration of 2 μM AS-DACA for four hours before they were examined for fluorescence. Cells were excited with a UV wavelength of 330 nm–385 nm in order to visualise AS-DACA accumulation in vesicles and a wavelength of 460 nm–490 nm to observe accumulation of the drug in the nucleus. Images were captured with a manual Olympus camera under a magnification of 600× and were used to produce tinted, composite images of both wavelength views.

**Figure 2:**
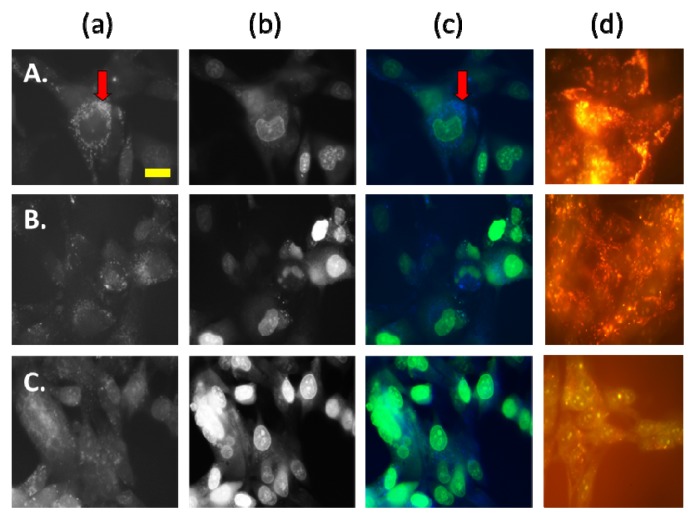
Fluorescence images of RD cells treated with (**A**) AS-DACA; (**B**) AS-DACA and Chlorpromazine (**C**); AS-DACA and 3-Methyladenine. Column (**a**) shows the signal following excitation at wavelengths of 330 nm–385 nm where AS-DACA produced blue fluorescence; Column (**b**) shows the signal following excitation at wavelengths 460 nm–490 nm where AS-DACA produced green fluorescence; Column (**c**) show the fluorescence images captured using true colour photography, tinted to produce a composite image of both filter views; Column (**d**) shows AO control experiment. Yellow bar = 10 μm.

**Figure 3:**
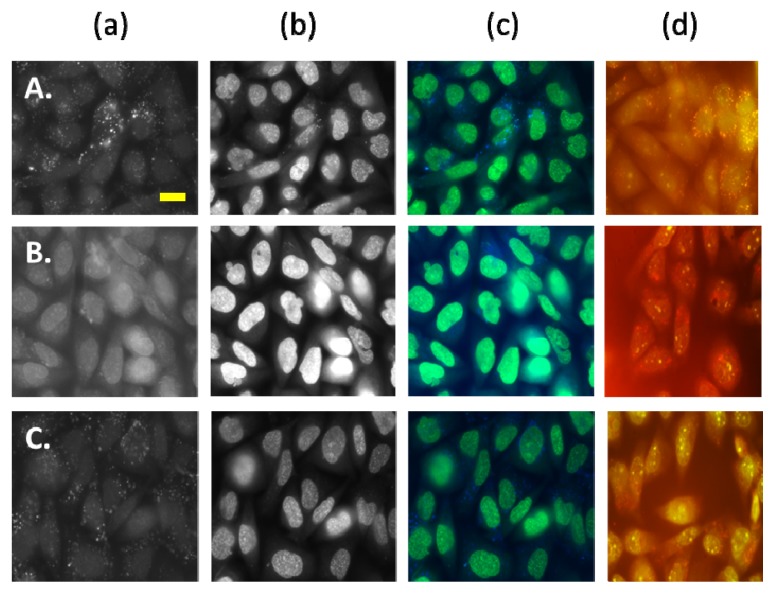
Fluorescence images of RH30 cells treated with (**A**) AS-DACA; (**B**) AS-DACA and Chlorpromazine; (**C**) AS-DACA and 3-Methyladenine. Column (**a**) shows the signal following excitation at wavelengths of 330 nm–385 nm where AS-DACA produced blue fluorescence; Column (**b**) shows the signal following excitation at wavelengths 460–490 nm where AS-DACA produced green fluorescence; Column (**c**) show the fluorescence images captured using true colour photography and tinted to produce a composite image of both filter views; Column (**d**) shows acridine orange (AO) control experiment. Yellow bar = 10 μm.

Following a four hour incubation with AS-DACA, both cell lines exhibited punctate blue vesicles dispersed in the cytoplasm along with an intense green fluorescence being observed in the nucleus of all cells indicating drug movement to the low pH environment of endocytic components as expected (Figures 2 and 3). The RD cell line displayed blue compartments, which were arranged in what appeared to be punctate ‘clusters’ of acidic organelles surrounding the nucleus (Figure 2 red arrows). Cells of the RH30 cell line however had blue vesicles that assumed a more random intracellular distribution and did not appear to converge with each other (Figure 3).

Spatial organisation of drug trapping endocytic compartments has implications in drug resistant phenotypes. A study by Jin *et al.* [36] characterised the distribution of daunorubicin in multiple leukaemia cell lines and found that vesicular drug accumulation was dependent on the spatial distribution of endosomes and microtubule networks. It was noted that in sensitive cells the endocytic compartments were evenly dispersed throughout the cytoplasm; which was also observed in sensitive RH30 cells. Endocytic clustering of resistant cells was related to a reduction in microtubule arrays which caused the juxtanuclear distribution of vesicles. Their spatial organisation allowed them to uptake more drug compared to sensitive counterparts and was the basis of their resistance phenotype. Since a similar pattern of endosomal organisation was noted in RMS cells, it is possible that the spatial dynamics of the endocytic compartment is implicated in AS-DACA sequestration.

Anticancer agents, particularly doxorubicin, also have fluorescence properties, which allow their distribution to be visually monitored by fluorescence microscopy [23,37,38]). AS-DACA retains the ability to be tracked visually and has structural properties that dictate its protonation according to the pH of the acidic compartments of the membrane trafficking system it is found. This study only provided qualitative data on AS-DACA sequestration. However, to ensure that the endocytic inhibitors were exerting their desired effect on the endocytic pathway, acridine orange (AO) was included and its intracellular distribution observed by fluorescence [39]. AO was added at a final concentration of 2 μM for 20 min and was observed under wavelengths of 490 nm–500 nm and 520 nm to visualize accumulation of orange vesicles and nuclear distribution respectively. Firstly, comparison of no treatment control images between RMS cell lines starkly illustrated the increased quantity of the orange vesicle fluorescence in the RD cell line compared to RH30 (Figures 2A(d) and 3A(d)). This notable result demonstrates the RD cell line as exhibiting lower baseline fluorescence intensity in the nucleus with no inhibitor treatment compared to RH30. This confirms the greater potential for vesicle sequestration in the RD cell line as compared to the RH30 as a general principle. To determine the influence of endocytic inhibitors on AS-DACA sequestration into intracellular compartments, the cell lines were treated with the MTD of Chlorpromazine and 3 Methyladenine for 68 h before they were exposed to a final concentration of 2 μM AS-DACA for four hours and examined for fluorescence and visualized as previously described.

#### 2.2.2. AS-DACA Intracellular Distribution with Chlorpromizine Co-Treatment in RMS Cell Lines

Multiple authors have described increased endocytic recycling and a greater number of endosomes to enhance drug sequestration in resistant cell lines, which serve as a protective mechanism of cytotoxic interference by anti-cancer drugs [32,38,40]. Treatment with the clathrin-mediated inhibitor Chlorpromazine was hypothesised to decrease the amount of endosomes by inhibiting the initiation of the endocytic pathway. Figures 2B and 3B illustrated the effect of Chlorpromazine treatment in RMS cell lines, reducing the amount of vesicles in both as shown by the panels (a). The corresponding accumulation of AS-DACA in the nucleus, as indicated by the panels (b), appeared more intense in RD cells, representing increased AS-DACA accumulation in the nucleus, leading to DNA damage and cytotoxicity. The sensitive cell line RH30 was less affected by Chlorpromazine treatment, with AS-DACA fluorescence in vesicles showing a reduction in the presence of the inhibitor [Figure 3B(a)], although this cell line had less vesicles prior to treatment and so the distinction in AS-DACA accumulation isn’t as profound as that observed in RD.

The resistant RMS cell line, RD, exhibits mechanisms of resistance that are more sensitive to the inhibitory effects of Chlorpromazine than RH30. These results taken together suggest RD employs methods of endocytic recycling or increased endosomal volume to improve the sequestration and associated resistance to AS-DACA.

#### 2.2.3. AS-DACA Intracellular Distribution with 3-Methyladenine Co-Treatment in RMS Cell Lines

3-Methyladenine is a class III PI3-kinase inhibitor that impedes the fusion of late endocytic compartments of the endosomal pathway [18,34,38,41]. AS-DACA sequestration intensification at this stage of membrane trafficking would be observed by a reduction of AS-DACA distribution into vesicles with addition of this agent. 3-Methyladenine affected intracellular accumulation of AS-DACA, most notably in the resistant RD cell line. Figures 2C and 3C illustrated the effect of 3-methyladenine on RD and RH30 respectively. Inhibitor effect on AS-DACA accumulation in RD was illustrated through an increased intensity of green fluorescence in the nucleus (Figure 2C(c)) compared to AS-DACA alone (Figure 2A(c)). The RH30 cells did not display the same shift in nuclear staining intensity for AS-DACA with 3-methyladenine treatment as observed in the resistant RD (Figure 3C(c)). This highlights the increased susceptibility of the resistant cell line to interference of the late endosomal compartment, suggesting that this compartment is enhanced to confer resistance. A reduction of AO accumulation into acidic compartments following 3-methyladenine treatment was observed by noting fewer orange vesicles surrounding the nucleus and a subsequent increase of green fluorescence within the nucleus was seen, highlighting the movement of AO to its nuclear target in the presence of 3-Methyladenine.

Reduction of AS-DACA sequestration in the presence of 3-methyladenine can be affiliated with prior findings that describe the 3-fold improved potency of AS-DACA in the presence of this inhibitor. Late endosomal and lysosomal compartments have been implicated in anti-cancer drug sequestration in multiple resistant cell lines [15,24,42–45] and based on the correlation of cytotoxicity and fluorescence data in this study, could also be a participant in AS-DACA resistance. Chapuy *et al.* [33] and Herlevsen *et al.* [46] have both illustrated active mechanisms of late endosomal compartments contributing to drug resistance phenotypes. Similarly, Hurwitz *et al.* [24] determined the lysosomal compartments contribution to drug resistance by enlargement of these compartments to encase more anti-cancer drug compared to sensitive cell lines. Taken together, our data indicates AS-DACA vesicle accumulation was more pronounced in RD compared to RH30 and was more susceptible to inhibitor treatments in the relatively resistant cell line RD. Visual observation of AS-DACA and AO accumulation in RMS cell lines highlighted the greater intensity of vesicle staining in the resistant RMS cell line, RD, with the accompanying reduction of nuclear accumulation. Hence, increased AS-DACA sequestration in the RD cell line is likely responsible for the resistance. RD cells were also more susceptible to treatment with inhibitors 3-methyladenine and chlorpromazine in relation to the reduction of AS-DACA vesicle accumulation as compared to RH30. These results highlight that an active mechanism of drug sequestration may be evident in RMS cells rather than that induced by diffusion through pH gradients.

### 2.3. Alterations to Endosomal Organelle Marker Proteins upon Treatment with AS-DACA or Endosomal Pathway Inhibitors

Several lines of evidence suggest that the observed sequestration of AS-DACA in RMS cells is caused by a redistribution mechanism which involves uptake into acidic vesicles of the membrane trafficking system. To confirm this, Wolf *et al.* [11] examined expression levels of markers specific for various vesicle types of the endocytic pathway. It was suspected that in an attempt to optimise AS-DACA redistribution from the nucleus, vesicular numbers may increase in resistant cells and that this could be reflected through examination of levels of early endosomal markers, EEA1 and Rab5 and late endosomal marker levels of Rab7 and LAMP1. The novel D52 protein marker was also included in the study. D52 is a tumour protein that is over-expressed in multiple cancers, however it has recently been established that it has a novel role in regulating membrane traffic [47]. In order to further define the vesicular sequestration mechanism in RMS, cell lines were treated with combinations of AS-DACA and the mentioned inhibitors. Levels of expected vesicles involved in AS-DACA drug redistribution following treatment could be assessed by detecting specific protein markers localised to endosomal and lysosomal membranes by Western Blot analysis.

#### 2.3.1. Effects of Endosomal Protein Expression in RMS Cells following AS-DACA and Chlorpromazine Treatment

**Figure 4:**
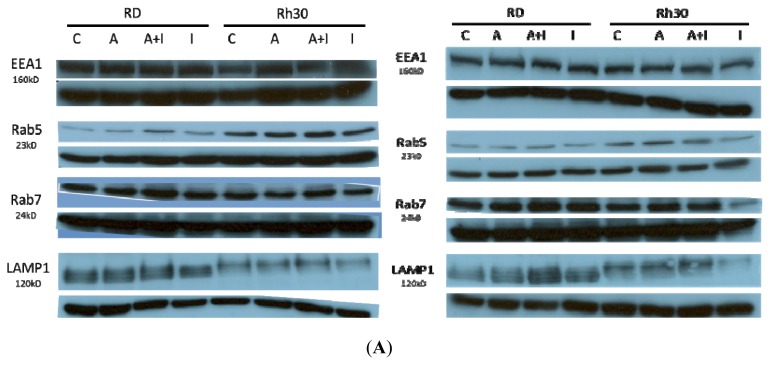

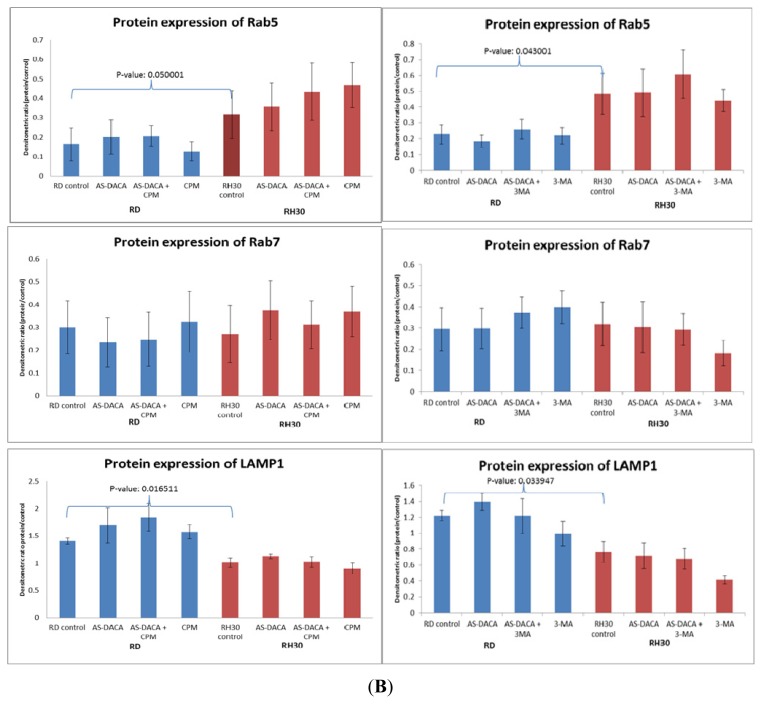
RMS cell lines were examined for endosomal protein expression by Western Blot analysis following AS-DACA treatment in the presence of Chlorpromazine (*left panel*) and 3 Methyladenine (*right panel*) inhibitors. (**A**) Representative autoradiograph bands for expression of EEA1, Rab5, Rab7 and LAMP1 (*upper bands*). Bands were quantified using Image J and the ratio with the alpha-tubulin loading control (*lower bands*) presented for Rab5, Rab7 and LAMP1 (**B**). Error bars represent the standard error from the mean of triplicate results. *p* value for the *t*-test comparison of RD *vs*. Rh30 untreated controls is shown. C = control (no treatment); A = AS-DACA; I = inhibitor.

Cell lines were treated with combinations of 1 × IC_50_ of AS-DACA and MTD of Chlorpromazine for 48 h to observe whether differences existed in endosomal marker (EEA1, Rab5, Rab7, LAMP1) expressions. All results are shown in the left panels of Figure 4. Early Endosomal Antigen 1 (EEA1) treatment is shown to have the same levels of expression between both cell lines and across all treatment combinations. The RH30 cell line expressed Rab5 to a greater extent compared to the RD cell line with an approximate 50% increase in intensity that was statistically significant (*p* = 0.05) according to two-tailed *t*-test. AS-DACA treatment induced an up-regulation of Rab5 in both cell lines. Chlorpromazine treatment induced a modest down-regulation of Rab5 in RD, while differentially inducing further up-regulation of Rab5 in RH30. Rab7 expression was shown to be relatively consistent between cell lines with no differential expression following treatment with AS-DACA and Chlorpromazine noted (Figure 4).

Lysosomal Associated Membrane-Protein-1 (LAMP1) is a marker of the late endosomal compartment [34,48]. LAMP1 expression levels were observed to be consistently higher in RD cells (Figure 4). The difference between LAMP1 expression in RD and RH30 no treatment controls was observed as being statistically different (*p* = 0.017) by evaluation of ratios with the two-tailed *t*-test. It was also noticed that in RD, treatment with both AS-DACA alone and AS-DACA and chlorpromazine treatment up-regulated the expression of LAMP1, which was then slightly lowered with treatment of chlorpromazine alone. This trend was also seen in RH30 but to a far lesser extent. Of most interest, LAMP1 proteins were located at different positions in the gel run for the two cell lines; RD was consistently of lower molecular weight compared to RH30. LAMP1 is known to be subject to post-translational protein alterations and is highly glycosylated at the amino-terminal side of its protein structure [44]. Glycosylation has been shown to influence protein trafficking and stability, but complete characterisation of this role has not yet been established [49]. Increased LAMP1 expression following AS-DACA treatment may therefore be a protective mechanism in RMS cells by inducing increased AS-DACA sequestration specifically in later endosomal compartments.

#### 2.3.2. Effects of Endosomal Protein Expression in RMS Cells Following AS-DACA and 3-Methyladenine Treatment

Both RMS cell lines were treated with combinations of 1 × IC_50_ AS-DACA and a MTD of 3-Methyladenine for 48 h and processed as before. Figure 4 (right panels) indicates that that EEA1 expression remained relatively constant across both cell lines and treatment types as previously found with treatment of chlorpromazine. Neither cell line exhibited statistically significant alterations in Rab5 expression following treatment with AS-DACA and/or 3-methlyadenine. This is further highlighted by the minimal changes in expression induced by endocytic inhibitor treatment with Chlorpromazine and 3-Methyladenine across both cell lines (Figure 4). Both these inhibitors induced a shift in sensitivity and intracellular distribution in the RD cell line by their apparent effect on sequestration events controlled by components of the endocytic pathway. Since inhibitor treatment did not affect early endosomal compartments, represented by EEA1 and Rab5 expression, we conclude that this early compartment is not involved in AS-DACA resistance.

A slight increase in Rab7 was observed upon co-treatment with AS-DACA and 3-methyladenine treatment and with 3-methyladenine treatment alone in RD cells, as illustrated in Figure 4. By contrast, 3 methyladenine caused a decrease in Rab7 band intensity in RH30 upon treatment. When RD was treated with AS-DACA and 3-methyladenine, the LAMP1 expression level was returned to that of the RD control, but when treated with 3-Methyladenine alone, expression was reduced below that of the level seen in previous treatments (Figure 4(B) right panel). LAMP1 expression was consistent in RH30 across most treatment types. However, like RD, treatment with 3-Methyladenine alone, reduced band intensity from approximately 20%–40%. (Figure 4B right panel).

LAMP1 expression levels in the resistant RMS cell line, RD, could be a contributing factor in the sequestration-induced resistance mechanism of AS-DACA. The late endosomal/lysosomal compartment was initially associated with AS-DACA resistance in RD (Figures 2 and 3) where inhibition of later endocytic organelle activity was accomplished with 3-methyladenine treatment which successfully reduced its intracellular accumulation. These findings collectively highlight a mechanism of late endosomal or lysosomal compartments in RMS resistance involving AS-DACA. The inclusion of lysosomal drug sequestration in cancer resistance has been widely associated with anti-cancer drug resistance in multiple studies. Late endosomal and lysosomal compartments have been shown to display a variety of mechanisms that take part in cellular resistance phenotypes including enlargement of lysosomal compartments, spatial arrangements favourable for drug uptake and active mechanisms to transport drugs directly into vesicles [12,33,45,46,48,50–52].

#### 2.3.3. Expression of D52 in RMS Cell Lines

**Figure 5:**
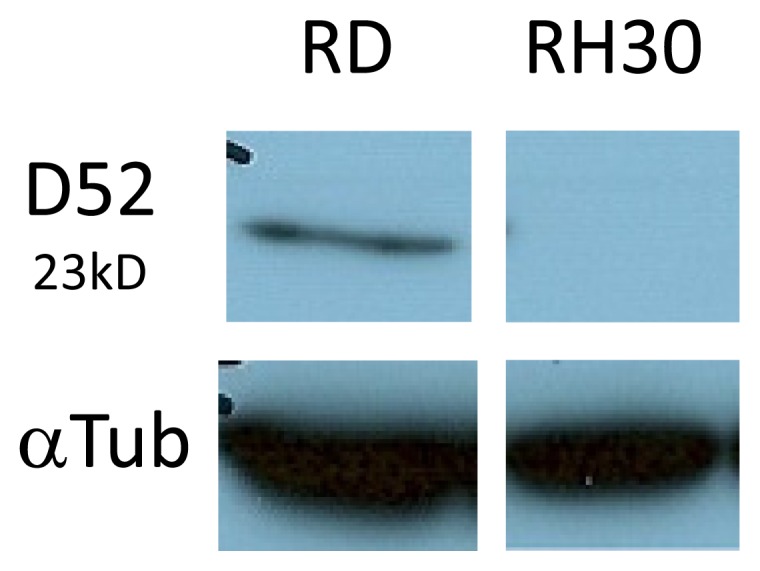
Western blot analysis of D52 in RMS cells; D52 has a band size of 23 kDa.

D52 expression has been associated with the regulation of lysosomal membrane protein trafficking to the plasma membrane [47], and so its exclusive expression in RD could potentiate a novel role in AS-DACA sequestration and resistance. An investigation carried out by Thomas *et al.* [47] established the expression of D52 in Chinese hamster ovary cells. They detailed D52’s co-localisation with markers restricted to the lysosome-like secretory vesicles: the adaptor protein AP-3, Rab27A, vesicle associated membrane protein, VAMP7, and LAMP1. D52 overexpression resulted in a discernible accumulation of LAMP1 on the plasma membrane and it was found its expression modulated the plasma membrane recycling of LAMP1 vesicles [47]. Figure 5 shows the expression levels of D52 across both RMS cell lines illustrating stark results with the obvious expression of D52 in RD, and its complete absence in RH30.

D52’s expression in the RD cell line further substantiates the involvement of the late endosomal/lysosomal compartment in AS-DACA resistance and its implied sequestration. Higher expression levels of LAMP1 in the resistant RMS cell line RD and its differential glycosylation between RMS cell lines associate the resistance of AS-DACA to the late endosomal compartment, possibly by increased AS-DACA sequestration in these organelles. These findings support the reduction in AS-DACA accumulation and cytotoxicity upon treatment with 3-Methyladenine. The exclusive expression of D52 in the RD cell line is an area of great interest and its role in AS-DACA resistance must be evaluated further.

## 3. Experimental Section

### 3.1. Drugs

Source, preparation and storage conditions for each drug used in the study were as follows. AS-DACA was obtained from Professor William Denny, Auckland Cancer Society Research Centre, NZ, made up with mQ-H_2_O to 10 mM, filter sterilised and stored at −20 °C. All endosomal inhibitors were obtained through Sigma, Australia. 3-Methyladenine was made up with DMSO to 10 mM, filter sterilised and stored at 4 °C in the dark Bafilomycin A1 was made up with DMSO to 500 μM and stored at −20 °C in the dark. Chloroquine was made up with 1× PBS to 10 mM, filter sterilised and stored at 4 °C in the dark. Finally, Chlorpromazine was made up with 1× PBS to 10 mM, filter sterilised and stored at 4 °C in the dark.

### 3.2. Cell Lines and Culture Conditions

The human RMS cell lines used in this investigation, RD and RH30 were obtained from the American Type Culture Collection, Virginia, USA. Cell lines were cultured and grown at 37 °C in humidified conditions with 5% CO2. RD was cultured in DMEM medium (Invitrogen, Carlsbad, CA, USA) and RH30 was cultured in RPMI medium (Invitrogen, Carlsbad, CA, USA), both were supplemented with 10% FBS (Sigma, St. Louis, MO, USA) with 1% glycerol added to the RPMI medium. Passage ratios from confluent plates were dependent on growth rates. Cells were removed from plates by aspirating media, washing once with sterile 1× PBS and incubating at 37 °C with 1 mL of 0.05% Trypsin-EDTA (ThermoFisher Scientific, Melbourne, Australia) for 5 min. Cell dilutions were seeded into new passages in sterile 10 cm plates with a total of 10 mL cell and media suspension.

### 3.3. Cytotoxicity Assay

Cells were passaged from a confluent plate as described above. Both cell lines had a minimum passage ratio split of 1:6 depending on cell confluency. 100 μL of diluted cells were pipetted using a multi-channel pipette into a 96 well plate and were allowed to grow at 37 °C and 5% CO_2_ for 24 h prior to drug treatment. Drug treatment was administered in serial dilutions (1:10) with drug dilutions made in fresh warmed media and aliquoted in 100 μL increments. AS-DACA dilutions were made to a final concentration range of 10 mM–1 fM, while the chemical inhibitors varied in starting dosages. Chlorpromazine was added at dilutions ranging from 500 μM to 50 fM, Bafilomycin A1 was added in the range 1 μM–0.1 fM, Chloroquine was added in the concentration range of 500 μM–50 fM and 3-Methyladenine was added at concentrations 5 mM–500 fM. The final well volume was always 200 μL of drug and cellular suspension. When AS-DACA and inhibitor treatment was administered, the inhibitor was added to each well at a maximum tolerated dose concentration and at a volume of 10 μL while AS-DACA was added at usual 1:10 serial dilution concentrations at a volume of 100 μL. To ensure the total well volume was consistently 200 μL across all treatments, only 90 μL of cell and media suspension was added for inhibitor and AS-DACA experiments. Each drug dilution had an assay repeat number of 4 for each plate and each drug treatment was repeated 3–5 times for each cell line. Following drug additions, the plates were incubated at 37 °C with 5% CO_2_ for 68 h; after this incubation 50 μL of 2 mg/mL (3-(4,5-dimethylthiazol-2-yl)-2,5-diphenyltetrazolium bromide (MTT) solution (Sigma-Aldrich, Sydney, Australia) was added to each well and incubated in the same conditions for four hours. MTT and media were aspirated from all wells and 100 μL of DMSO was added to dissolve the crystallised formazan at the bottom of each well. The intensity of the resultant solution was determined using a Labsystems Multiskan Ascent multi-well plate spectrophotometer at 540 nm.

Averages of the quadruplicate dilution repeats were calculated and taken as percentages of the “no treatment” wells to determine cell growth, this was recorded as % cell viability. Calculated percentages of at least triplicate repeat experiments were analysed with GraphPad prism 4 to generate sigmoidal curves. Non-linear regression, dose response functions were utilised to obtain an IC_50_ value for each drug treatment which signifies the concentration of drug required to inhibit cell viability by 50% over the total 72 h of drug exposure.

### 3.4. Fluorescence Microscopy

#### 3.4.1. AS-DACA Distribution

The intracellular distribution of AS-DACA was determined by utilising its fluorescent properties with epifluorescent microscopy. Cells were passaged from confluent plates as described above and grown on autoclaved coverslips in a 6 well plate and incubated at 37 °C with 5% CO_2_ overnight before a final concentration of 2 μM of AS-DACA was added and incubation for 4 h. The plates were then covered with foil to reduce photo-bleaching and incubated for a further 4 h. When co-treated with inhibitors, cells were treated for a period of 68 h and then incubated with AS-DACA for 4 h as above. Media was aspirated and coverslips washed once with 1× PBS. The coverslip was immediately mounted onto a glass slide with FluorSave mounting solution and placed in a light proof container. The slide was viewed on an Olympus BX50 epifluorescent microscope under a 60× objective lens and oil. Initial UV excitation (330–385 nm) was achieved using a U-MWV filter and allowed fluorescence of blue vesicles to be observed. A U-MWIBA3 filter was used for AS-DACA excitation within the range of 460–490 nm with resulting green emission detected between 510 and 550 nm. Images were captured using a Jenoptik ProgRes CF Scan digital camera plus true colour photography function and ProgRes^®^ V2.5 software. Images of both filter views were used to create composite images which were tinted according to emission spectra using the mentioned digital software.

#### 3.4.2. Acridine Orange

Acridine Orange (Sigma-Aldrich, Sydney, Australia) solution (40 mM) was added to cells grown on autoclaved coverslips in a 6 well plate at a final concentration of 2 μM which were incubated in the dark for 20 min at 37 °C. Cells which had prior treatment with inhibitors were incubated for 68 h before AO addition and control no treatment cells were grown at these same conditions. Media was aspirated and coverslips washed once with 1× PBS. The coverslip was immediately mounted onto a glass slide with FluorSave mounting solution and placed in a light proof container. The slide was viewed as on an Olympus BX50 epifluorescent microscope under a 60× objective lens and oil. AO distribution was monitored by first performing excitation at 490–500 nm using a U-MWIBA3 and was then viewed at 520 nm using a U-MWIB filter. Images were captured as described above.

### 3.5. Western Blotting

#### 3.5.1. Protein Extraction, SDS-PAGE and Transfer

Cells were grown on 6 well plates. Cell lines were treated with AS-DACA at their respective IC_50_ and maximum tolerated dose concentration of inhibitors Chlorpromazine and 3-Methyladenine at a total of 48 h. Media was then aspirated and cells were washed twice in ice cold 1× PBS and placed on ice. Cells were lysed by addition of SDS in 1 M Tris (pH 8.5) supplemented with protease inhibitors to the plate. A Cell lifter was used to dislodge cells which were then pipetted into an eppendorf tubewioth the subsequent lysate then sonified and centrifuged at 13,000 RPM at 4 °C. Protein supernatant was then collected in an eppendorf tube and stored at −80 °C. The bicinchoninic acid (BCA assay) determined protein concentrations as per manufacturer’s instructions and read on a Labsystems Multiskan Ascent multi-well plate spectrophotometer measured the intensity of single well solutions at 570 nm. The protein concentrations were established by obtaining a mean of triplicate wells and using the gradient equation of the standard curve.

SDS-polyacrylamide gel electrophoresis (SDS-PAGE) on 12.5% or 7.5% gels was carried out using protein extracts ranging from 20 to 30 μg per well. Protein lysates were mixed with 5× loading buffer (50% glycerol, 15% 1 M Tris, pH 6.8, 8.5% 2-Mercaptoethanol, 0.01% Brilliant Blue) in a 4:1 ratio and boiled at 95 °C for 5 min in order to denature the proteins. 15 μL of Benchmark pre-stained protein ladder (Invitrogen (Life Technologies), Melbourne, Australia) was loaded onto the gel alongside samples. Samples were run for 30 min at 80 V then increased to 120 V for 90 min.

Filter paper and sponges were allowed to soak in 1× transfer buffer (25 mM Tris, 250 mM Glycine, 20% Methanol, 0.005% SDS) while a polyvinylidene fluoride (PVDF) membrane was rehydrated in methanol and then rinsed in 1× transfer buffer. The gel was kept hydrated with transfer buffer and placed in the transfer cassette with the sponges and PVDF and then placed in the transfer tank (Bio-Rad, Sydney, Australia) with freshly made 1× transfer buffer. The tank was immersed in ice and an electrical current of 80 V was run through the transfer tank for 2 h. On completion of the transfer, membranes were placed in methanol and allowed to air dry for 15 min before being rinsed in methanol again and rinsed in water. Membranes were then stained with 0.1% Ponceau S in glacial acetic acid for 2 min to confirm transfer quality, washed twice with water before being placed in plastic slips. Membranes were stored in TBS (100 mM Tris-HCl, 150 mM NaCl) at 4 °C for a maximum of 2 days before immunostaining.

#### 3.5.2. Immunostaining

The following primary antibodies were used at the following concentration. Alpha-Tubulin mouse monoclonal (Sigma-Aldrich, Sydney, Australia) at 1:300. EEA1 mouse monoclonal (Abcam, Sapphire Bioscientific, Sydney, Australia) at 1:200. Rab5C babbit polyclonal (Sigma-Aldrich, Sydney, Australia) at 3:1000 and Rab7 mouse monoclonal (Abcam, Sapphire Bioscientific, Sydney, Australia) 3 μg/mL. LAMP1 rabbit polyclonal (Abcam, Sapphire Bioscientific, Sydney, Australia) 2 μg/mL. The D52 rabbit polyclonal antibody was generously provided by Assoc. Prof. J. Byrne The Children’s Hospital at Westmead, Sydney and was used at 1:50 dilution.

Membranes were blocked with 5% dry skim milk in TTBS (0.1% Tween-20 in TBS) either for 1 h on a shaking platform at room temperature or overnight at 4 °C. Membranes were washed 3 times for 5 min each in TTBS and then sealed in a plastic slip with primary antibody diluted with 5% skim milk and TTBS (blocking solution). Membranes were incubated with primary antibodies on a shaking platform for 2 h at room temperature. Membranes were washed a further 3 times (10 min each) in TTBS before incubating with appropriate secondary antibody in a plastic slip for 1 h on a shaking platform at RT. Donkey anti-rabbit horseradish peroxidise-conjugated secondary antibody (GE Healthcare Life Sciences, Sydney, Australia) was diluted at a ratio of 1:10,000 with blocking solution while the donkey anti-mouse secondary antibody (GE Healthcare Life Sciences, Sydney, Australia) was diluted at a concentration of 1:5000 with blocking solution. Following secondary antibody incubation, membranes underwent 3 × 10 min washes in TTBS and exposed to Western Lightning chemiluminescent reagent (Perkin Elmer, Melbourne, Australia) for 5 min. Excess solution was removed and membranes were placed in plastic strips in preparation for protein band exposure. Membranes were exposed to X-ray film for time periods ranging from 1 min to an overnight exposure, dependent on signal strength. X-ray films were developed using the automatic Konica SRX-101 X-ray processor. Membranes were stored at −4 °C in sealed plastic slips. Image J was employed to quantify band intensities by densitometry and loading differences were accounted for by comparison to an alpha-tubulin loading control.

## 4. Conclusions

RMS cells displayed a differential cytotoxic response to AS-DACA, with the RD cell line being 24-fold less sensitive to treatment in comparison to the RH30 cell line. Previous studies carried out by Wolf *et al.* [11] suggested AS-DACA is sequestered into acidic organelles of the membrane trafficking system. We hypothesize that the decreased sensitivity to the drug AS-DACA in RMS cells is due to sequestration of the drug into acidic vesicles of the endosomal pathway.

Acidification inhibitors bafilomycin A1 and chloroquine did not alter the cytotoxic profile nor intracellular distribution of AS-DACA suggesting that sequestration and resistance could not be explained by the well-established hypothesis of weak base drug sequestration according to the pH partitioning mechanism. Further, endosomal expression levels observed suggested the early endosomal compartment was not involved in AS-DACA resistance and instead that the late endosomal/lysosomal compartment was implicated.

LAMP1 is a marker of the late endosomal and lysosomal compartment and was expressed at higher levels in the relatively resistant RMS cell line RD in comparison to the sensitive counterpart cell line RH30. LAMP1 also demonstrated an interesting pattern in its band morphology; it appears to be differentially glycosylated between cell lines. This may be associated with a differential ability of cell lines to collect AS-DACA into lysosomes as a mechanism of resistance. Tumour marker protein D52 displayed an interesting result in that it was expressed exclusively in RD and had nil expression in RH30. This also provides linkage of AS-DACA resistance and sequestration to the late endosomal compartment as D52 is involved in lysosomal trafficking [47]. Further, the late endosomal/lysosomal inhibitor 3-methyladenine induced a 3-fold increase of AS-DACA sensitivity in the RD cell line and also induced a redistribution of AS-DACA from endocytic compartments, as observed by fluorescence microscopy.

Combined, these findings implicate the late endosomal compartment as being associated with AS-DACA resistance in RMS cells. The clathrin-mediated endocytosis inhibitor. Chlorpromazine doubled the sensitivity of the RD cell line to AS-DACA, suggesting that endosomal volume and recycling mechanisms contribute to AS-DACA resistance. It is possible that multiple mechanisms simultaneously contribute to the resistance phenotype observed in RMS cell lines. Studies by Gong *et al.* [53] highlighted the ability of separate endosomal mechanisms to contribute to a single resistance phenotype; they established distinct roles for the golgi apparatus and lysosomes in the sequestration of drugs in the multidrug-resistant human leukemic cell line. Our results suggest that AS-DACA is sequestered into RMS cells by an active mechanism most likely enhanced at the late endosomal/lysosomal compartment.

## Figures and Tables

**Table 1:** Comparison of cytotoxic response (IC_50_ μM) in RMS cells.

Treatment	RD	RH30	Fold change	*T*-test
AS-DACA	0.92 ± 0.047	0.039 ± 0.007	23.5	<0.05 *
Chlorpromazine	39.3 ± 1.11	50.6 ± 9.1	0.78	-
Bafilomycin A1	0.0015 ± 0.0003	0.0013 ± 0.0002	1.18	-
Chloroquine	9.61 ± 0.24	8.94 ± 3.0	1.07	-
3-Methyladenine	823 ±178	692 ± 262	1.19	-

**AS-DACA plus**				
Chlorpromazine	0.51 ± 0.053		1.81	0.008 *
		0.038 ± 0.0095	1.03	0.771
Bafilomycin A1	1.45 ± 0.34		0.63	0.315
		0.054 ± 0.0084	0.72	0.132
Chloroquine	0.96 ± 0.55		0.96	0.562
		0.053 ± 0.0084	0.74	0.425
3-Methyladenine	0.31 ± 0.20		2.97	0.033 *
		0.041 ± 0.0073	0.95	0.803

* Statistically significant fold change (*p* < 0.05).
